# Psychometric evaluation of the Diabetes Injection Device Experience Questionnaire (DID-EQ) and Diabetes Injection Device Preference Questionnaire (DID-PQ)

**DOI:** 10.1186/s41687-018-0064-3

**Published:** 2018-09-19

**Authors:** Louis S. Matza, Katie D. Stewart, Rosirene Paczkowski, Karin S. Coyne, Brooke Currie, Kristina S. Boye

**Affiliations:** 1 Patient-Centered Research, Evidera, 7101 Wisconsin Avenue, Suite 1400, Bethesda, MD 20814 USA; 2 Eli Lilly and Company, Indianapolis, IN USA

**Keywords:** Type 2 diabetes, Injection device, GLP-1 receptor agonist, Patient-reported outcomes measures, PRO, Psychometric validation

## Abstract

**Background:**

Previous research has examined patient perceptions of insulin injection devices. However, a range of injectable medications other than insulin are now used to treat type 2 diabetes. No patient-reported outcome (PRO) instruments have been developed taking into account the perceptions of patients using newer injection devices, which are often different from those used in the past. Therefore, the primary purpose of this study was to evaluate a new PRO instrument focusing on patients’ experiences with injection devices, including those used for newer treatments such as GLP-1 receptor agonists.

**Methods:**

Patients with T2D treated with non-insulin injectable medications were recruited via advertisements and six clinical sites in the US. All participants completed the draft Diabetes Injection Device - Experience Questionnaire (DID-EQ) and additional measures administered for validity assessment. Participants who had experience with two non-insulin injection devices also completed the draft Diabetes Injection Device - Preference Questionnaire (DID-PQ). Analyses focused on item reduction (item performance, exploratory factor analysis), reliability, and validity.

**Results:**

One hundred fourty two patients (mean age = 63.0y; 56.3% female) participated. Item reduction yielded a 10-item version of the DID-EQ, including a 7-item Device Characteristics subscale and three global items assessing satisfaction, ease of use, and convenience of the injection device. The DID-EQ demonstrated good internal consistency reliability (Cronbach’s alpha of Device Characteristics subscale = 0.80) and 7-day test-retest reliability (ICCs: 0.92 for Device Characteristics subscale; 0.65 to 0.91 for the three global items). Construct validity was demonstrated via correlations with previously validated instruments (e.g., correlations with the DTSQ treatment satisfaction subscale ranged from 0.56 to 0.60, all *p* < 0.0001; correlations with the TRIM-D Device ranged from 0.63 to 0.77, all p < 0.0001). Descriptive analyses of the DID-PQ were conducted with a subset of 27 participants who were able to use it to compare two devices.

**Conclusions:**

This psychometric evaluation supports the reliability and validity of the DID-EQ, while providing initial information on the performance of the DID-PQ. These brief questionnaires complement measures of treatment efficacy and provide a more thorough picture of patients’ experiences with non-insulin injectable treatments for type 2 diabetes.

**Electronic supplementary material:**

The online version of this article (10.1186/s41687-018-0064-3) contains supplementary material, which is available to authorized users.

## Background

Patient-reported outcome measures (PROs) can provide important insight into the experience of patients with type 2 diabetes. A range of PRO measures have been developed to assess overall treatment satisfaction and perceptions of the insulin injection process in this patient population [1–4]. However, injectable medications other than insulin are now used to treat type 2 diabetes, primarily medications in the class of glucagon-like peptide-1 (GLP-1) receptor agonists [5, 6]. PRO instruments designed to assess perceptions of insulin treatment are not necessarily well-suited for newer treatments such as the GLP-1 receptor agonists, which often differ from insulin in multiple aspects of treatment administration and the injection device.

Although the GLP-1 receptor agonists tend to have similar efficacy and safety [7–11], the medications in this class vary in their injection devices and treatment administration requirements. The devices used to inject the GLP-1 receptor agonists vary in size, requirements for needle handling, and multiple versus single use [12–17]. In addition, patients are required to reconstitute some of the medications within these devices prior to the injection [13, 15], while other GLP-1 receptor agonists do not require this preparation [12, 14, 16–18]. Dose frequency also differs among these medications, as some are injected every day [12, 16, 17] while others are injected once weekly [13–15, 18].

These attributes of injection devices and the injection process could have an impact on patients’ quality of life and preference among non-insulin injectable treatments. Therefore, a pair of draft PRO measures was recently developed to assess perceptions of non-insulin injection devices. Content validity of the two draft measures was supported by qualitative research with a total of 52 patients treated with non-insulin injectable devices for type 2 diabetes (32 concept elicitation interviews to generate the items, followed by 20 cognitive interviews focused on refining the initial draft instruments) [19, 20]. Each draft questionnaire included 20 items derived directly from perceptions of patients in the qualitative interviews.

The first questionnaire was designed to assess perceptions of a single injection device, while the second asks patients to report preferences between two devices. Despite some content overlap with previously developed PRO measures, these two new instruments diverge from the insulin-focused measures in several ways. The two new instruments omit concepts that would be irrelevant to patients receiving many of the non-insulin medications (e.g., small dose adjustments, using the device in public). In addition, they include concepts that are specifically relevant to non-insulin injectable devices (e.g., variations in dose frequency, flexibility with regard to dose timing, requirements for needle handling). The primary purpose of the current study was to perform item reduction followed by the first psychometric evaluation of the Diabetes Injection Device Experience Questionnaire (DID-EQ). The secondary purpose was to begin examining the Diabetes Injection Device Preference Questionnaire (DID-PQ) in the subgroup of patients who had been treated with multiple non-insulin injection devices and were therefore able to report preferences between two devices.

## Methods

### Study design

This study was the first quantitative assessment of the DID-EQ and DID-PQ. Study participants were treated with non-insulin injectable medications for type 2 diabetes (either a GLP-1 receptor agonist or pramlintide). All participants completed the 20-item draft version of the DID-EQ, and patients who had been treated with more than one non-insulin injectable medication in the past 12 months also completed the 20-item draft version of the DID-PQ. One-third of the sample was randomized to complete the DID-EQ and the DID-PQ (if applicable) a second time 7 ± 2 days after the first questionnaires were completed so that test-retest reliability could be evaluated. Data analysis began with item reduction in order to derive streamlined versions of the questionnaires that could be administered efficiently with minimal patient burden. Then, the shortened final versions of the questionnaires were examined in terms of reliability and validity.

### Participants

Participants were required to be (1) currently residing in the US; (2) at least 18-years-old at the time of enrollment; (3) diagnosed with type 2 diabetes by a recognized medical professional; (4) currently receiving treatment with a non-insulin injectable medication [with or without insulin] for type 2 diabetes and be able to report/recall characteristics of the injection device; (5) able to provide proof of current non-insulin injectable medication [either a GLP-1 receptor agonist or pramlintide]; (6) able to read, speak, and understand English; (7) able and willing to give written informed consent prior to study entry; and (8) able to complete protocol requirements. Patients were recruited via newspaper and online advertisements in a wide range of geographical locations within the US (Birmingham, AL; Huntsville, AL; Mobile, AL; Washington, DC; Atlanta, GA; Louisville, KY; Boyd/Greenup, KY; Carter, KY; Lawrence, KY; New Orleans, LA; Baton Rouge, LA; Acadiana, LA; Kansas City, MO; Charlotte, NC; Columbia, SC; Nashville, TN; Houston, TX; San Antonio, TX; Dallas, TX; and Charleston, WV), as well as through six clinics in West Palm Beach, FL; Port Charlotte FL; Anderson, SC; Honolulu, HI; Alexandria, VA; and Wooster, OH.

Efforts were made to ensure that the sample included patients who had used the full range of non-insulin injectable medications for type 2 diabetes that were available when the study was conducted. All participants were required to provide proof of medication prior to the study assessment in order to confirm their eligibility. Acceptable forms of proof included a scan or photo of the medication, injection device, injection device packaging, prescription with the participant’s name, or a doctor’s note with the participant’s name. Proof of medication was provided via mail, fax, email, or text message.

### Measures

In addition to a demographic and clinical form, participants completed the DID-EQ, DID-PQ, and two other instruments that were used to examine the validity of the DID-EQ.

#### Diabetes injection device experience questionnaire (DID-EQ)

The diabetes injection device questionnaires (DID-EQ and DID-PQ) were developed to assess patients’ experiences with diabetes injection delivery systems. The draft versions of the questionnaires administered in the current study each had 20 items with parallel content assessing injection device experience and preference. The DID-EQ was designed to assess patients’ experiences with a single injection device. For each item, respondents report perceptions of their injection device by selecting a choice from a 4-point Likert response scale (ranging from “strongly disagree” to “strongly agree,” “not at all confident” to “completely confident,” “very dissatisfied” to “very satisfied,” or “very difficult” to “very easy”). Participants are instructed to select an answer based on how they “currently” feel about their injection device.

The recall period is an important aspect of a PRO instrument [21]. The DID instruments were not designed to quantify health status over a given period of time. Instead, these questionnaires were intended to assess current perceptions of injection devices. Therefore, the instructions do not ask respondents to remember past sentiments about injection devices or attempt to average their perceptions over a period of time. The two questionnaires ask only about current perceptions/preferences related to injection devices. In the DID-EQ instructions, participants are told to “Please select one response for each item to indicate how you currently feel about the device used to inject your non-insulin medication for diabetes.”

#### Diabetes injection device preference questionnaire (DID-PQ)

The DID-PQ asks patients to indicate their preference between two injection devices using items parallel to those on the DID-EQ. This instrument was completed by only the subset of participants who had received treatment with more than one non-insulin injectable medication in the 12 months prior to completing study measures. For each item, respondents indicate whether they strongly prefer device 1, prefer device 1, strongly prefer device 2, prefer device 2, or have no preference. At the top of columns for the response options, patients indicate which treatment is device 1 and which is device 2. The instructions for the DID-PQ ask respondents to “Please select only one response for each item to indicate which of the two injection devices you prefer.”

#### Diabetes treatment satisfaction questionnaire (DTSQ)

The DTSQ assesses patient satisfaction with diabetes treatment [22, 23]. This patient-reported outcome measure has a total of eight items, with a recall period of “the past few weeks” and is intended for use among patients with type 1 or type 2 diabetes. It consists of a six-item scale assessing treatment satisfaction and two items assessing perceived frequency of hyperglycemia and hypoglycemia. Items are scored on a scale from 0 to 6, and the treatment satisfaction total score is computed by adding responses to the six items within the scale, yielding scores with a possible range from 0 to 36, with higher scores indicating greater treatment satisfaction. The status version of the questionnaire (i.e., not the change version) was administered in the current study.

#### Treatment related impact measure – Diabetes device (TRIM-D device)

The TRIM-D Device assesses the impact of diabetes treatment devices on functioning and well-being [24, 25]. This self-administered, patient-reported measure has a total of eight items, with a recall period of “over the past two weeks” and is intended for use among patients with type 1 or type 2 diabetes. Items are rated on a five-point scale with response options ranging from 1 (“not at all”) to 5 (“extremely”), and are grouped within two domains assessing Device Bother and Device Function. The TRIM-D Device is scored for each domain, with higher scores indicating more positive perceptions.

### Data collection procedures

The study protocol and procedures were reviewed and approved by an institutional review committee (Ethical & Independent Review Services IRB, December 23, 2015, Protocol 15156–01). Data collection occurred from January to May of 2016. Eligible participants were scheduled for a study assessment to be conducted by telephone with a trained member of the study team. Prior to the scheduled study assessment, each participant was sent a packet of study materials by mail including an informed consent form and study questionnaires (DID-EQ, DID-PQ, TRIM-D Device, DTSQ, demographic and clinical form). A member of the study team called the participant at the scheduled time to administer informed consent, answer questions about the study, and provide instructions for independently completing the questionnaires. Participants returned completed consent forms and questionnaires by mail.

One-third of participants were randomly assigned to complete a retest assessment 7 ± 2 days after the initial assessment. Participants assigned to the retest subgroup were mailed a second packet including another DID-EQ and (if they had experience with a second non-insulin injection device) a DID-PQ. Similar to the initial assessment, the retest assessment began with a phone call in which a study staff member instructed the participant to complete and return the questionnaire(s).

### Statistical analysis procedures

Analyses were performed with SAS version 9.4 (SAS Institute, Cary, NC) and MPlus (Version 7).

#### Item reduction

Item reduction was performed based on analysis of the DID-EQ. Decisions made based on the DID-EQ were also applied to item reduction with the DID-PQ, resulting in two parallel versions of the diabetes injection device questionnaire assessing the same constructs, one questionnaire assessing patient experience with one device and another assessing preference between two devices. Decisions regarding item reduction and subscale identification were made based on a range of factors, specified a priori. Items were considered for deletion if they met any of the following criteria: (1) ceiling or floor effect (i.e., items where more than 80% of the sample endorsed the highest or lowest response options); (2) greater than 5% missing responses; (3) redundancy as indicated by a particularly high correlation with another item (*r* > 0.85); (4) lack of relationship to other items as indicated by Cronbach’s alpha with the item deleted (i.e., if alpha increased by > 10% upon deletion of an item, then that item would have been considered for possible exclusion because it could potentially reduce internal consistency of the scale); and (5) items with insufficient factor loading (i.e., < 0.40) or loading on multiple factors in the factor analysis described below. Additionally, when deciding whether to delete any item, the clinical importance of each item was also considered, based on qualitative research conducted to support development of the questionnaires prior to this psychometric study [19, 20]. Items were not necessarily deleted based on any individual statistical criterion or cut-off. Instead, decisions about item reduction were based on consideration of all these factors.

Exploratory factor analysis (EFA) was performed on the full 20-item draft, the 17 items not including the three global items, and additional shortened versions after dropping items. Both orthogonal (assumes uncorrelated factors) and oblique (assumes factors are correlated) rotations were used to examine the underlying structure among the items (i.e., the extent to which items group together) using principal axis factoring extraction. The number of factors was initially set to no factors (i.e., nfactor = 0) to allow the number of factors to be determined based on Eigen values (with 1.0 as an approximate cut-off value, depending on item content and value) and the scree plot.

The EFAs were also used to determine whether the items should be grouped into multiple subscales and scored accordingly. The final three items of the DID instruments each assessed a global concept. These three items were intended to stand alone and be scored independently from the other items so that overall satisfaction, ease of use, and convenience of injection devices could be assessed and reported separately. Therefore, these items were not considered for deletion during the item reduction process, and they were not considered for inclusion in a subscale or total score. Instrument scoring, including the strategy for handling missing data, was finalized after item reduction and subscale identification were completed.

#### Psychometric evaluation

After item reduction and subscale development, analyses were conducted to examine reliability and validity of the final versions of the DID-EQ and DID-PQ. Internal consistency reliability was assessed for the multi-item scale of each instrument using Cronbach’s formula for coefficient alpha. Cronbach’s alpha values greater than 0.70 are generally considered to be acceptable for PROs [26–28].

Test-retest reliability of the multi-item scale and three global items of the DID instruments was examined using data from the subgroup of participants in the retest sample. For the DID-EQ, intraclass correlation coefficients (ICC) were conducted to evaluate the degree of association between the two assessments, and paired t-tests were used to examine whether there were statistically significant score changes between the two assessments. Standards for test-retest reliability vary, but ICCs of over 0.60 or 0.70 are usually thought to indicate adequate reliability [27–29]. It was also hypothesized that no significant change would be seen between the baseline and retest DID-EQ scores. ICCs and t-tests were not computed for the DID-PQ due to the small sample size of patients who completed this instrument twice (*n* = 11).

For the DID-EQ, convergent validity was examined via Spearman correlations with previously developed questionnaires assessing related constructs (TRIM-D Device and DTSQ). Known-groups validity was examined by categorizing patients based on responses to these other instruments, and then comparing DID-EQ scores among these subgroups of patients using t-tests or analyses of variance (ANOVAs). Additional exploratory descriptive analyses were conducted to examine the extent to which the DID-EQ performed as expected in subgroups of patients categorized by GLP-1 receptor agonist treatments with different injection devices.

Whereas the DID-EQ could be examined using generally accepted methods for analyzing validity, the DID-PQ is not well-suited for typical analysis of convergent and known-groups validity. The DID-PQ is not a conventional PRO instrument because its response options do not range from low to high on the construct being assessed. Instead, each item assesses preference, with stronger preference for one injection device over another at opposite ends of the scale and a neutral response in the middle of the scale. Therefore, correlations with previously developed instruments assessing treatment satisfaction would not be appropriate. However, to provide initial insight into construct validity, DID-PQ responses were examined with respect to whether respondents reported preferring their current or previous treatment. It was hypothesized that respondents would tend to prefer their current treatment.

## Results

### Sample description

A total of 189 patients were screened for eligibility, 158 met eligibility criteria and were scheduled for a study assessment. Of the 158, 14 could not be reached or rescheduled, and two others did not complete key study measures (e.g., the DID-EQ), resulting in a per protocol sample of 142 participants. Of the 142, 47 were randomized to the retest subgroup, and 42 of these individuals completed the retest assessment. Of the 142 participants, 27 provided DID-PQ data for the first assessment, and 13 of these 27 participants were randomized to the retest assessment, which was completed by 11 participants.

The mean age (SD) of the sample was 63.0 (9.8) years (Table 1). More than half the sample was female (56.3%), while a majority was white (75.4%) and married (69.0%). Most participants were either retired (56.3%) or employed full-time (34.5%). Mean age (SD) at the time of diabetes diagnosis was 49.6 (12.3) years. Patients were treated for type 2 diabetes with a wide range of non-insulin injectable medications (Table 1). Many participants (73.2%) received treatment with oral medication, while fewer than half (40.8%) were treated with insulin. The most frequently reported comorbid medical conditions were hypertension (66.2%), arthritis (38.0%), and heart attack or heart disease (25.4%). The demographic and clinical characteristics of the retest sample were similar to those of the total sample (Table 1).

**Table 1:** Demographic and Clinical Characteristics

Characteristic	Total Sample (*N* = 142)	Retest Sample (*N* = 42)
Age (mean, SD)	63.0 (9.8)	65.1 (8.4)
Gender (n, %)		
Male	62 (43.7%)	19 (45.2%)
Female	80 (56.3%)	23 (54.8%)
Ethnic background (n, %)		
Hispanic or Latino	4 (2.8%)	2 (4.8%)
Not Hispanic or Latino	138 (97.2%)	40 (95.2%)
Racial background (n, %)		
Asian	12 (8.5%)	2 (4.8%)
Black	22 (15.5%)	6 (14.3%)
Caucasian	107 (75.4%)	32 (76.2%)
Other^a^	7 (4.9%)	3 (7.1%)
Marital status (n, %)		
Single	11 (7.7%)	3 (7.1%)
Married/Living with partner	98 (69.0%)	30 (71.4%)
Other^b^	33 (23.2%)	9 (21.4%)
Employment status (n, %)^c^		
Full-time work	49 (34.5%)	15 (35.7%)
Part-time work	7 (4.9%)	1 (2.4%)
Other^d^	86 (60.6%)	26 (61.9%)
Education level (n, %)		
University degree or more	70 (49.3%)	20 (47.6%)
No university degree	72 (50.7)	22 (52.4%)
Current medication (n, %)^f^		
Non-insulin injectable medication^g^	**142 (100.0%)**	**42 (100.0%)**
Exenatide extended release pen	26 (18.3%)	8 (19.0%)
Exenatide extended release tray	12 (8.5%)	1 (2.4%)
Exenatide	13 (9.2%)	4 (9.5%)
Pramlintide	2 (1.4%)	1 (2.4%)
Albiglutide	14 (9.9%)	5 (11.9%)
Dulaglutide	38 (26.8%)	15 (35.7%)
Liraglutide	38 (26.8%)	8 (19.0%)
Oral medication	**104 (73.2%)**	**30 (71.4%)**
Blimepiride	16 (11.3%)	6 (14.3%)
Glipizide	13 (9.2%)	3 (7.1%)
Canagliflozin	9 (6.3%)	3 (7.1%)
Metformin	83 (58.5%)	24 (57.1%)
Other	29 (20.4%)	9 (21.4%)
Insulin	**58 (40.8%)**	**18 (42.9%)**
Insulin lispro	8 (5.6%)	4 (9.5%)
Insulin detemir	16 (11.3%)	3 (7.1%)
Insulin aspart	8 (5.6%)	3 (7.1%)
Insulin glargine	27 (19.0%)	7 (16.7%)
Other^h^	13 (9.2%)	6 (14.3%)

^a^ Other racial background includes for total sample includes American Indian (*n* = 2), Euro-American (*n* = 1), European (*n* = 1), Hawaiian (*n* = 2), Pacific Islander (Hawaiian) (*n* = 1). Other racial background for retest sample includes American Indian (*n* = 1), Euro-American (*n* = 1), and Pacific Islander (Hawaiian) (*n* = 1)

^b^ Other marital status for total sample includes divorced (*n* = 15), separated (*n* = 3), widowed (*n* = 14), and life partner (*n* = 1). Other marital status for retest sample includes divorced (*n* = 4), separated (*n* = 2)

^c^ Not mutually exclusive

^d^ Other for total sample includes employment status includes homemaker/housewife (*n* = 11), student (*n* = 2), unemployed (*n* = 4), retired (*n* = 80), disabled (*n* = 9), self-employed (*n* = 2); self-employed entrepreneur (*n* = 1); work from home (*n* = 1). Other for retest sample includes self-employed (*n* = 1)

5 Other education status for per protocol sample includes associates degree (*n* = 3), J.D. (*n* = 1), R.T license (*n* = 1), vocational degree – law enforcement certification (*n* = 1)

^e^ Participants were permitted to list multiple current medications and multiple discontinued medications. Therefore, the sub-rows do not necessarily add to the bolded total rows

^f^ One participant (ID 122–068) reported currently taking two non-insulin injectable medications for treatment of type 2 diabetes (pramlintide and exenatide extended release tray). When completing the DID-EQ, the participant rated pramlintide. When completing the DID-PQ, the participant compared pramlintide (Device 1) and exenatide extended release tray (Device 2)

^g^ Other for total sample includes: pioglitazone (*n* = 5); dapagliflozin (*n* = 5); empagliflozin (*n* = 5); sitagliptin and metformin (*n* = 4); sitagliptin (*n* = 3); repaglinide (*n* = 2); nateglinide (*n* = 2); glyburide (*n* = 1); saxagliptin (*n* = 1); linagliptin (*n* = 1). Other for retest sample includes: dapaglifozin (*n* = 2); empagliflozin (*n* = 2); nateglinide (*n* = 2); sitagliptin and metformin (*n* = 1); sitagliptin (*n* = 1); repaglinide (*n* = 1)

^h^ Other for total sample includes: Unspecified insulin (*n* = 5); insulin degludec (*n* = 3); human insulin (*n* = 2); insulin glargine (*n* = 2); insulin glulisine (*n* = 1). Other for retest sample includes: insulin unspecified (*n* = 3); human insulin (*n* = 1); insulin glulisine (*n* = 1); insulin degludec (*n* = 1)

### Item reduction

As specified a priori, item reduction was performed based on analysis of the DID-EQ. Decisions made based on the DID-EQ were then applied to the DID-PQ, resulting in two parallel versions of the questionnaire. EFAs were initially run to identify multi-factor solutions. However, these analyses did not appear to yield multi-factor solutions with clear and conceptually distinct factors. In addition, there was a large decrease from the first to the second eigenvalue. Therefore, it was decided to proceed with a single factor solution and to delete items to derive a briefer instrument that would minimize patient burden while focusing on the best performing items.

A total of 10 items were dropped from the draft DID-EQ during the item reduction process. Three items were dropped due to ceiling effects. Seven additional items were dropped because of low factor loading on the single factor, as well as conceptual differences from the other items in the scale. Dropping items with lower factor loadings resulted in improved model fit. Factor loadings from the EFA of the seven retained items in the Device Characteristics scale are presented in Table 2 (range: 0.49 to 0.67). A confirmatory factor analysis (CFA) was run in Mplus with the final seven items assessing device characteristics, and the comparative fit index (CFI) was 0.958.

**Table 2:** DID-EQ: Descriptive Statistics and Exploratory Factor Analysis

Item	N	Mean	SD	Range	Floor (%)^a^	Ceiling (%)^a^	EFA Factor Loadings^b^
1. How difficult is it to prepare the injection device and medication for use?	142	3.7	0.6	1.0–4.0	1 (0.7%)	101 (71.1%)	0.5418
2. How difficult is it to fit the injection into your routine?	142	3.6	0.6	2.0–4.0	0 (0.0%)	95 (66.9%)	0.6105
3. How difficult is it to bring the injection device with you when it is necessary to inject away from home?	140	3.3	0.7	2.0–4.0	0 (0.0%)	67 (47.9%)	0.4858
4. How confident are you that the injection device provides the correct dose of medication every time?	142	3.3	0.8	1.0–4.0	2 (1.4%)	70 (49.3%)	0.6548
5. How confident are you that you are using the injection device correctly?	141	3.4	0.7	2.0–4.0	0 (0.0%)	78 (55.3%)	0.6654
6. How satisfied are you with the size of the needle?	142	3.5	0.8	1.0–4.0	4 (2.8%)	90 (63.4%)	0.6612
7. How satisfied are you with the time it takes to prepare and inject each dose of medication?	141	3.6	0.7	1.0–4.0	2 (1.4%)	103 (73.0%)	0.5769
Device Characteristics Subscale	142	83.0	15.8	38.1–100.0	0 (0.0%)	33 (23.2%)	–
8. Overall, I am satisfied with the injection device.	142	3.5	0.6	1.0–4.0	1 (0.7%)	81 (57.0%)	–
9. Overall, it is easy to use the injection device.	142	3.5	0.6	2.0–4.0	0 (0.0%)	84 (59.2%)	–
10. Overall, it is convenient to use the injection device.	142	3.5	0.6	2.0–4.0	0 (0.0%)	80 (56.3%)	–

^a^ These percentages indicate the percent of patients at the floor and ceiling of the possible range of each item

^b^ Exploratory factor analysis (EFA) of the seven items included in the Device Characteristics subscale of the final DID-EQ (1-factor solution with oblique rotation)

### Final versions and scoring of the DID-EQ and DID-PQ

In sum, item reduction resulted in a final 10-item version of the DID-EQ for rating experiences with a single injection device. Each item is rated on a four-point scale with higher scores indicating more positive perceptions of injection device characteristics. The first seven items assess patient perceptions of specific characteristics of injection delivery systems, and these seven items comprise the Device Characteristics scale with a possible score range of 0 to 100 (higher scores indicating more positive perceptions of device characteristics). To compute the subscale score, the seven individual item scores are first summed, resulting in a raw score with a possible range from 7 to 28. Then, for ease of interpretation, the raw score is transformed onto a scale with the possible range from 0 to 100 using the following formula: (actual raw score – lowest possible raw sum score) / possible raw score range × 100. For this scale, if < 50% of the seven items are missing, the subscale score may be computed with the mean of the answered items used to impute a score for the missing items. If > 50% of the items are missing, no subscale score should be calculated, and the subscale score should be considered missing.

The final three items of the DID-EQ are global items assessing overall satisfaction, ease of use, and convenience of injection devices. Because these items assess three distinct global concepts, they are each scored separately on a 1 to 4 scale with higher scores indicating more positive perception of the injection device. For a group of patients completing the DID-EQ, the mean score for each global item may be reported, as well as the frequency of patients within each of the four response categories. For the global scores, data should not be imputed. If a response to one of these three items is missing, it is not possible to derive a score for that item. Thus, when reporting results for the DID-EQ, four scores should be reported: the Device Characteristics scale score and the three global item scores for satisfaction, ease of use, and convenience. The final version of the DID-EQ is presented in Additional file 1, and a detailed scoring guide is available upon request.

The 10 items dropped for the DID-EQ were also dropped for the DID-PQ, resulting in a questionnaire for assessing patient preferences between two injection devices with regard to the same 10 items included on the DID-EQ. On the DID-PQ, each item is rated on a five-point scale allowing patients to indicate whether they prefer or strongly prefer one of the devices over the other. For each item, patients may also respond by selecting the “no preference” (i.e., neutral) response option, indicating that they have no preference between the two devices. Importantly, the response options of the DID-PQ do not range from high to low on a single dimension. Therefore, it is currently recommended that mean scores are not reported for the five response options of the DID-PQ. Instead, it is recommended that DID-PQ results be reported descriptively and categorically as the frequency and percentage of patients reporting each response to each item. In the current study, the DID-PQ items were treated as continuous items only for the calculation of Cronbach’s alpha for the seven-item Device Characteristics scale. The final version of the DID-PQ is presented in Additional file 2.

### Descriptive statistics

Mean scores on the 10 DID-EQ items were in the upper range of the scale, ranging from 3.3 to 3.7, and the mean score for the Device Characteristics scale was 83.0 (Table 2). For each of the 10 items, 47.9% to 73.0% selected the top response option indicating positive perceptions of the injection device. For five of the items, patients in this sample used the full range of the scale (i.e., response options 1 to 4), while for the other five items, patients provided responses of 2, 3, or 4. On the DID-PQ, patients tended to report preferences for their current device over previous devices (Table 3).

**Table 3:** Preference Questionnaire: Frequencies and Percentages for Each Response Option (*N* = 27)

	Current Device			Previous Device	
Item	Strongly Prefer	Prefer	No Preference	Prefer	Strongly Prefer
1. Ease of preparing the injection device and medication for use	14 (51.9%)	5 (18.5%)	2 (7.4%)	1 (3.7%)	5 (18.5%)
2. Ease of fitting the injection into your routine	16 (59.3%)	4 (14.8%)	3 (11.1%)	1 (3.7%)	3 (11.1%)
3. Ease of bringing the injection device with you when it is necessary to inject away from home	8 (29.6%)	8 (29.6%)	7 (25.9%)	3 (11.1%)	1 (3.7%)
4. Confidence that the injection device provides the correct dose of medication every time	8 (29.6%)	8 (29.6%)	7 (25.9%)	3 (11.1%)	1 (3.7%)
5. Confidence that you are using the injection device correctly	12 (44.4%)	5 (18.5%)	8 (29.6%)	2 (7.4%)	0 (0%)
6. The size of the needle	11 (40.7%)	5 (18.5%)	7 (25.9%)	1 (3.7%)	3 (11.1%)
7. The time it takes to prepare and inject each dose of medication	12 (44.4%)	4 (14.8%)	5 (18.5%)	2 (7.4%)	4 (14.8%)
8. Overall satisfaction with the injection device	13 (48.1%)	5 (18.5%)	2 (7.4%)	5 (18.5%)	2 (7.4%)
9. Overall ease of using the injection device	15 (55.6%)	3 (11.1%)	3 (11.1%)	3 (11.1%)	3 (11.1%)
10. Overall convenience of using the injection device	17 (63.0%)	3 (11.1%)	2 (7.4%)	3 (11.1%)	2 (7.4%)

### Reliability

#### DID-EQ

The DID-EQ Device Characteristics scale had a Cronbach’s alpha of 0.80, indicating good internal consistency reliability. Dropping any individual item resulted in a slight decrease in alpha (alpha ranging from 0.76 to 0.79, depending on which item was dropped). Test-retest reliability of the DID-EQ was assessed using data from the 42 participants who completed the measure at both assessments. ICCs were 0.92 for the Device Characteristics subscale, 0.65 for the satisfaction item, 0.91 for the ease of use item, and 0.76 for the convenience item. Based on these ICCs, the DID-EQ generally demonstrated good test-retest reliability. Paired t-tests found no statistically significant change in any DID-EQ scale between the two timepoints.

#### DID-PQ

The Device Characteristics subscale of the DID-PQ demonstrated good internal consistency reliability, with a Cronbach’s alpha of 0.90, and dropping any individual item resulted in a slight change (0.86 to 0.93, depending on which item was dropped).

### Validity

#### DID-EQ

The DID-EQ scales demonstrated convergent validity as indicated by Spearman correlations with TRIM-D Device and DTSQ scales (Table 4). Correlation coefficients ranged from 0.47 to 0.77, which are in the medium to large range [30]. Known-groups validity of the DID-EQ was evaluated by comparing scores of participants categorized based on their response to DTSQ item 1 (How satisfied are you with your current treatment?). The DTSQ item has a response scale ranging from 0 to 6 with higher scores indicating greater treatment satisfaction. For the ANOVA presented in Table 5, participants were categorized into four groups: DTSQ item 1 responses of 6, 5, 4, and < 3. Groups indicating greater satisfaction on the DTSQ item consistently had higher scores on the DID-EQ, with most pairwise comparisons between groups being statistically significant. Similar analyses in which patients were categorized based on item 4 of the DTSQ (How convenient have you been finding your treatment to be recently?) and the TRIM-D Device total score yielded similar findings.

**Table 4:** Convergent Validity: Spearman Correlations of the DID-EQ with TRIM-D Device and DTSQ Measures

	Correlations with TRIM-D Device Subscales			Correlations with DTSQ
DID-EQ Subscales	Total Score (*N* = 140)	Device Function (*N* = 141)	Device Bother (*N* = 140)	
Device Characteristics	0.77	0.67	0.65	0.60
Global Items				
Satisfaction	0.69	0.60	0.57	0.56
Ease of Use	0.65	0.60	0.49	0.57
Convenience	0.63	0.59	0.47	0.58

**Table 5:** Known-Groups Validity of the DID-EQ: ANOVA by Item #1 of the DTSQ

	DTSQ Item #1: How satisfied are you with your current treatment?^a^				
DID-EQ	6 (*n* = 67) Mean (SD)	5 (*n* = 67) Mean (SD)	4 (*n* = 27) Mean (SD)	<=3 (*n* = 13) Mean (SD)	Pairwise Comparisonp values^b^
Device Characteristics	89.1 (12.9)	82.4 (14.0)	73.5 (16.5)	72.5 (18.5)	A: 0.19; B: 0.0001; C: 0.0035; D: 0.13; E: 0.22; F: 0.10
Global Items					
Satisfaction	3.78 (0.42)	3.40 (0.74)	3.26 (0.59)	2.85 (0.69)	A: 0.02; B: 0.0018; C: < 0.0001; D: 0.82; E: 0.03; F: 0.21
Ease of Use	3.76 (0.46)	3.60 (0.55)	3.19 (0.62)	2.92 (0.64)	A: 0.56; B: 0.0001; C: < 0.0001 D: 0.03; E: 0.0024; F: 0.55
Convenience	3.76 (0.46)	3.49 (0.56)	3.22 (0.58)	3.00 (0.58)	A: 0.099; B: 0.0002; C: < 0.0001 D: 0.28; E: 0.046; F: 0.66

^a^ Higher DTSQ scores indicate greater satisfaction

^b^ANOVA with Scheffe’s post-hoc comparisons of DID-EQ scores between subgroups of participants categorized based on scores on DTSQ item #1 (A: DTSQ item #1 score of 6 vs. 5; B: 6 vs. 4; C: 6 vs. <=3; D: 5 vs. 4; E: 5 vs. <=3; F: 4 vs. <=3)

Although the purpose of this study was not to differentiate between treatments, and the sample size does not offer sufficient power to detect statistically significant differences between treatment groups, descriptive analyses of treatment subgroups offer additional support for known-groups validity of the DID-EQ. Across the total sample, patients rated seven different medications on the DID-EQ. Of these seven medications subgroups, the lowest mean scores were for medications with injection devices and procedures that would generally be considered less convenient. For example, the medication administered with a vial and syringe had mean scores of 66.9, 3.09, 3.27, and 3.27 for the Device Characteristics scale and three global items assessing satisfaction, ease of use, and convenience, respectively (*n* = 11). Scores for a GLP-1 receptor agonist administered in an injection pen, but requiring extensive preparation (e.g., attaching the needle, reconstituting the medication) had scores in a similar range (74.2, 3.15, 3.31, 3.31; *n* = 26). DID-EQ scores were higher for GLP-1 receptor agonists with simpler injection procedures, such as a weekly injection requiring no medication preparation or needle handling (89.5, 3.74, 3.79, 3.71; *n* = 38). Although these results should be interpreted with caution due to the small sample size within each medication subgroup, differences between medication groups are in the direction that would be expected based on injection device characteristics.

#### DID-PQ

Responses to all DID-PQ items indicate that participants tended to prefer their current injection device over previously discontinued devices (Table 3). This suggests that, in this small subgroup, the DID-PQ yielded findings in the expected direction, providing preliminary support for validity. Because the DID-PQ does not have response scales that range from low to high on a given construct, typical analyses of construct validity comparing this instrument to previously validated instruments are not applicable.

## Discussion

This psychometric evaluation supports the use of the DID-EQ for assessing patient perceptions of non-insulin injection devices. Item reduction resulted in the final 10-item DID-EQ, including three global items and the seven-item Device Characteristics scale assessing specific characteristics of the injection device and injection experience. The three global items quantify overall satisfaction, ease of use, and convenience of the injection devices, while the Device Characteristics scale provides an indication of specific injection device features that may contribute to these overall perceptions. Measurement properties of the questionnaire were supported with results suggesting good test-retest reliability, internal consistency reliability (of the multi-item Device Characteristics scale), convergent validity, and known-groups validity. Furthermore, minimal missing data in this validation study add to qualitative results from previously reported cognitive interviews supporting the instrument’s ease-of-use and comprehensibility. Overall, these results suggest that the DID-EQ would be a useful tool in studies examining the patient experience with non-insulin injectable medications for type 2 diabetes.

The DID-EQ addresses a gap in PRO measures for the assessment of treatment experiences among patients with type 2 diabetes. The previous measures are either general instruments [31] or questionnaires specifically targeted toward insulin treatment [1, 2, 4, 24, 25, 32–36]. The general instruments, such as the DTSQ [31], may be used with a broad range of treatments, but they do not yield specific information on injection devices. While the insulin focused questionnaires have items on injections, they were not designed or validated to assess experiences with commonly used newer injectable treatments including the GLP-1 receptor agonists. Therefore, these previous questionnaires omit key features that differentiate among the newer devices (e.g., ease of preparing the medication/device), while including items that are not applicable to most GLP-1 receptor agonists. For example, the TRIM-D Device questionnaire [24, 25] includes the item “How easy is it to adjust your medication for small dose changes?” Most of the available GLP-1 receptor agonist devices do not require or allow for dose selection or changes, which makes this item irrelevant and potentially confusing for many patients using non-insulin injection devices. Of the 102 patients in the current study who were using devices that do not allow for dose adjustment, 24 chose not to answer this item, 29 selected the response at the ceiling, and 28 selected the response at the floor. The high rate of missing data and extreme responses at contradictory ends of the scale suggests that this item could be problematic in a sample of patients treated with GLP-1 receptor agonists. In contrast, the DID-EQ was derived directly from the input of patients who have experience with the newer non-insulin injectable treatments, and therefore, it includes the issues most relevant to this patient population, without including concepts that would be irrelevant to patients not treated with insulin.

In this validation study, mean DID-EQ scores were in the upper range of the scale, suggesting that patients tended to have positive perceptions of their injection devices. Still, there was some variability in responses. Mean scores for subgroups using more convenient injection pens were higher than those for subgroups with less convenient injection procedures. For example, mean scores for GLP-1 receptor agonist devices on the ease of use global item ranged from 3.27 to 3.79. Although this difference was in the expected direction, it is not known whether a difference of 0.52 on this four-point scale should be considered clinically meaningful or important to patients. Therefore, future research is needed to identify the magnitude of difference between devices on the DID-EQ subscale and global items that can be considered meaningful to patients.

When comparing two injectable treatments that both have positive scores on the DID-EQ, one way to determine if one injection device is truly preferable over the other from the patient perspective would be to administer the DID-PQ. This questionnaire allows patients to directly compare two injection devices and indicate whether they have a strong preference, a preference, or no preference between the two devices on a range of characteristics. It is possible that patients could believe that two devices are acceptable (which could yield similarly high scores on the DID-EQ), but strongly prefer one over the other (which would be reflected on the DID-PQ). Therefore, the DID-PQ could be useful in head-to-head comparisons between treatments, particularly when the goal is to differentiate between injection devices that are both likely to be perceived as acceptable by patients.

The current study provides initial support for the DID-PQ, but the subgroup of patients in this study who could compare two non-insulin injection devices was small. Furthermore, analysis of reliability and validity of the DID-PQ is not straightforward because of the unusual structure of the items (i.e., ranging from strongly prefer one device to strongly prefer the other device). For assessment of DID-PQ reliability, the use of Cronbach’s alpha is questionable at this time because DID-PQ item results are presented categorically (e.g., the frequency and percentage of respondents who strongly prefer one device over another), rather than as summed continuous scores. Still, Cronbach’s alpha is presented in this study because it is the only currently available indication of DID-PQ reliability (because so few of the retest participants completed the DID-PQ). In light of this limitation, the data on DID-PQ internal consistency reliability should be interpreted with caution, although future research with a larger sample of patients completing the DID-PQ may be able to derive and examine a continuous scoring approach for this instrument.

With regard to validity of the DID-PQ, it was encouraging that responses reflected a consistent preference for patients’ current devices over their previous devices. This pattern of results suggests that, in this subgroup of 27 patients completing the DID-PQ, the instrument generally performed as expected. As with reliability, research with larger samples is needed to further support the validity of the DID-PQ for comparison of non-insulin injection devices.

Additional limitations of the DID instruments and this psychometric evaluation study should be acknowledged. As with the development of any PRO instrument, there was a trade-off between comprehensiveness and brevity. The DID questionnaires were intended to be brief so that they could be included in clinical or observational studies without adding substantial patient burden. Therefore, while the DID instruments capture global perceptions of injection devices along with detailed assessment of some features, these instruments cannot provide a completely comprehensive assessment of the injection experience.

Furthermore, the applicability of the DID-EQ and DID-PQ to devices beyond those used for the currently available non-insulin injectable medications is unknown. For example, new injectable medications could be developed with unique device features that are not captured by these instruments. Alternatively, it is also possible that the questionnaires could be useful for assessing a broader range of devices such as those used for insulin or perhaps combination treatments involving both insulin and a GLP-1 receptor agonist. Future research can examine content validity and psychometric properties of the DID questionnaires for use with additional injection devices beyond those used for the currently available GLP-1 receptor agonists.

Another limitation is that the sample size of this initial validation study is not sufficient for subgroup analyses with significance testing and therefore, the extent to which the DID-EQ can differentiate among treatment groups is not known. Confidence in PRO measures develops over time based on psychometric data across multiple studies [37]. Current results for these new instruments are encouraging, and future research can further examine measurement properties, while beginning to derive guidelines for interpreting differences in scores.

In sum, this psychometric evaluation study adds to previous qualitative research [19, 20] supporting the use of the DID-EQ for assessing patient perceptions of non-insulin injection devices and the injection process. In addition, the DID-PQ may also be useful in situations when patients can compare two non-insulin injection devices. In clinical and observational studies, these brief questionnaires can complement measures of treatment efficacy and provide a more thorough picture of patients’ experiences with non-insulin injectable treatments for type 2 diabetes.

## Additional files

Additional file 1: Diabetes Injection Device Experience Questionnaire (DID-EQ). (DOCX 21 kb)

Additional file 2: Diabetes Injection Device Preference Questionnaire (DID-PQ). (DOCX 19 kb)

## Abbreviations

*ANOVA*: Analyses of variance
*CFA*: Confirmatory factor analysis
*CFI*: Comparative fit index
*DID*: Diabetes Injection Device
*DID-EQ*: Diabetes Injection Device Experience Questionnaire
*DID-PQ*: Diabetes Injection Device Preference Questionnaire
*DTSQ*: Diabetes Treatment Satisfaction Questionnaire
*EFA*: Exploratory factor analysis
*GLP-1*: Glucagon-like peptide-1
*ICC*: Intraclass correlation coefficients
*PRO*: Patient-reported outcome
*T2D*: Type 2 diabetes
*TRIM-D Device*: Treatment-Related Impact Measures for Diabetes and Diabetes Devices
*TRIM-D*: Treatment-Related Impact Measures for Diabetes
*UK*: United Kingdom
*US*: United States
