# Supplementary material for: Psychometric evaluation of the Diabetes Injection Device Experience Questionnaire (DID-EQ) and Diabetes Injection Device Preference Questionnaire (DID-PQ)
Source: J Patient Rep Outcomes. 2018 Sep 19;2:44. doi: 10.1186/s41687-018-0064-3 (PMC6153201; doi:10.1186/s41687-018-0064-3)
Supplement: Supplementary file 1 — Diabetes Injection Device Experience Questionnaire (DID-EQ). (DOCX 21 kb) [file 41687_2018_64_MOESM1_ESM.docx]

**Additional File 1: Diabetes Injection Device Experience Questionnaire (DID-EQ)**

Please select one response for each item to indicate how you currently feel about the device used to inject your medication for diabetes.

If you take more than one injectable medication for diabetes, you should answer the questions about **only one** of the medication devices.

Please complete this questionnaire thinking about the device used to inject: ______________________

*Name of medication*

|  | **Very difficult** | **Somewhat difficult** | **Somewhat easy** | **Very easy** |
| --- | --- | --- | --- | --- |
| 1. How difficult is it to prepare the injection device and medication for use? | □ | □ | □ | □ |
| 1. How difficult is it to fit the injection into your routine? | □ | □ | □ | □ |
| 1. How difficult is it to bring the injection device with you when it is necessary to inject away from home? | □ | □ | □ | □ |
|  | **Not at all confident** | **Somewhat confident** | **Very confident** | **Completely confident** |
| 1. How confident are you that the injection device provides the correct dose of medication every time? | □ | □ | □ | □ |
| 1. How confident are you that you are using the injection device correctly? | □ | □ | □ | □ |
|  | **Very dissatisfied** | **Somewhat dissatisfied** | **Somewhat satisfied** | **Very satisfied** |
| 1. How satisfied are you with the size of the needle? | □ | □ | □ | □ |
| 1. How satisfied are you with the time it takes to prepare and inject each dose  of medication? | □ | □ | □ | □ |
|  | **Strongly disagree** | **Disagree** | **Agree** | **Strongly agree** |
| 1. Overall, I am satisfied with the  injection device. | □ | □ | □ | □ |
| 1. Overall, it is easy to use the  injection device. | □ | □ | □ | □ |
| 1. Overall, it is convenient to use the injection device. | □ | □ | □ | □ |
