# Supplementary material for: Psychometric evaluation of the Diabetes Injection Device Experience Questionnaire (DID-EQ) and Diabetes Injection Device Preference Questionnaire (DID-PQ)
Source: J Patient Rep Outcomes. 2018 Sep 19;2:44. doi: 10.1186/s41687-018-0064-3 (PMC6153201; doi:10.1186/s41687-018-0064-3)
Supplement: Supplementary file 2 — Diabetes Injection Device Preference Questionnaire (DID-PQ). (DOCX 19 kb) [file 41687_2018_64_MOESM2_ESM.docx]

**Additional file 2: Diabetes Injection Device Preference Questionnaire (DID-PQ)**

Please select **only one** response for each item to indicate which of the two injection devices you prefer.

The names of the two medications should be written in the spaces provided below.

|  | **Device 1:**  *Name of Medication* | |  | **Device 2:**  *Name of Medication* | |
| --- | --- | --- | --- | --- | --- |
|  | **Strongly prefer  device 1** | **Prefer device 1** | **No preference** | **Prefer device 2** | **Strongly prefer device 2** |
| 1. Ease of preparing the injection device and medication for use | □ | □ | □ | □ | □ |
| 1. Ease of fitting the injection into your routine | □ | □ | □ | □ | □ |
| 1. Ease of bringing the injection device with you when it is necessary to inject away  from home | □ | □ | □ | □ | □ |
| 1. Confidence that the injection device provides the correct dose of medication every time | □ | □ | □ | □ | □ |
| 1. Confidence that you are using the injection device correctly | □ | □ | □ | □ | □ |
| 1. The size of the needle | □ | □ | □ | □ | □ |
| 1. The time it takes to prepare and inject each dose of medication | □ | □ | □ | □ | □ |
| 1. Overall satisfaction with the injection device | □ | □ | □ | □ | □ |
| 1. Overall ease of using the injection device | □ | □ | □ | □ | □ |
| 1. Overall convenience of using the injection device | □ | □ | □ | □ | □ |
